# Comparison of the Effects of Oral Midazolam and Intranasal Dexmedetomidine on Preoperative Sedation and Anesthesia Induction in Children Undergoing Surgeries

**DOI:** 10.3389/fphar.2021.648699

**Published:** 2021-12-15

**Authors:** Yu-Hang Cai, Cheng-Yu Wang, Yang Li, Jia Chen, Jun Li, Junzheng Wu, Hua-Cheng Liu

**Affiliations:** ^1^ Department of Anesthesiology, Perioperative and Pain Medicine, The Second Affiliated Hospital and Yuying Children’s Hospital of Wenzhou Medical University, Key Laboratory of Anesthesiology of Zhejiang Province, The Second Affiliated Hospital and Yuying Children’s Hospital of Wenzhou Medical University, Wenzhou, China; ^2^ Department of Anesthesiology, Cincinnati Children’s Hospital, Cincinnati, OH, United States

**Keywords:** dexmedetomidine, midazolam, premedication, intranasal, oral

## Abstract

**Background and Purpose:** Premedication with either oral midazolam or intranasal dexmedetomidine prior to surgery remains less than ideal. The aim of this study was to investigate whether the combination of those two drug regimens would have any beneficial effects on the preoperative sedation and the children’s compliance during anesthesia inhalation induction.

**Experimental Approach:** One hundred thirty-eight children aged 2–6 years were randomly allocated into three groups: Group M with oral midazolam 0.5 mg kg^−1^, Group D with intranasal dexmedetomidine 2 μg kg^−1^, and Group M + D with intranasal dexmedetomidine 1 μg kg^−1^ plus oral midazolam 0.5 mg kg^−1^. The primary outcome was the children’s compliance during inhalation induction with sevoflurane. The secondary outcomes included the preoperative sedative effects, behavior scores, parental separation anxiety scores, and the postoperative incidence of emergence agitation and recovery time.

**Results:** Subjects in Group M + D showed higher satisfaction scores of compliance (*p* = 0.0049) and mask acceptance (MAS) (*p* = 0.0049) during anesthesia inhalation induction. Subjects in Group M + D had a significantly shorter time than those in Groups M and D to achieve the desired sedation level (*p* < 0.001) and remained at a higher sedation score in the holding area and up to the anesthesia induction after drug administration (*p* < 0.001).

**Conclusion and Implications:** We conclude that pediatric patients premedicated with intranasal dexmedetomidine 1 μg kg^−1^ plus oral midazolam 0.5 mg kg^−1^ had significantly improved anesthesia induction compliance, and quicker onset to achieve and maintain a satisfactory level of sedation than those premedicated separately with two drugs. Therefore, the combined premed regimen is a greater choice when we are expecting a higher quality of sedation and a smoother anesthesia induction in children undergoing the surgeries.

## Introduction

Preoperative anxiety remains a vexing issue, and it exists in nearly 50% of pediatric patients (Kain et al., 1999; Chorney and Kain, 2009). The inhalation induction of anesthesia is also the most distressing moment a child could experience during the perioperative period. Children who are extremely anxious prior to surgery or/and have gone through a rough inhalation induction of anesthesia, if not addressed appropriately, are most likely to develop untoward clinical consequences, including intraoperative hemodynamic changes and abnormal cardiac excitability, anesthesia emergence delirium and long-term postoperative sleep disturbance, and other negative behaviors. Various factors like parental separation, unfamiliar surroundings, and fear of doctors and syringe needles can provoke children’s anxiety before surgery (Kain et al., 2004a; Kain et al., 2004b; Davidson and McKenzie, 2011), and then, poor compliance with anesthesia induction would ensue. Therefore, pediatric anesthesiologists have very challenging tasks to minimize child’s anxiety and to improve their anesthesia induction compliance prior to surgery.

Many pharmacological and non-pharmacological methods have been attempted to alleviate anxiety and improve compliance with anesthesia induction, such as sedative premedication, the presence of parents, and training programs for participants and their parents. Among the pharmacological options, midazolam has been one of the most popular premedications used today (Kain et al., 2004a; Kain et al., 2004b), and it has shown to be more effective than parental presence or placebo in reducing anxiety and improving compliance during anesthesia induction (Kain et al., 1998; Kain et al., 2000). A study has shown that oral midazolam 0.5 mg.kg-1 could reduce anxiety at both moments of parental separation and induction of anesthesia (Cox et al., 2006). However, only 70% of children accept oral midazolam well (Khalil et al., 2003), and some possible adverse effects of midazolam, such as paradoxical reaction and negative postoperative behavioral changes, have confounded its beneficial effects (Kanegaye et al., 2003; Khalil et al., 2003). Therefore, the technique with single oral midazolam may not be as good as clinicians highly expected in efforts to ease the preoperative anxiety and improve the smoothness of inhalation induction in children undergoing surgery.

Dexmedetomidine is a highly selective alpha‐2 adrenoceptor agonist, and it stimulates adrenergic receptors in locus coeruleus to induce a state of natural sleep with analgesic and anti-anxiety properties (Jannu et al., 2016; Feng et al., 2017). Intranasal dexmedetomidine has been increasingly used for its effectiveness of sedation and enhanced bioavailability profile (Miller et al., 2018; Wolbold et al., 2003). Yuen et al. demonstrated that intranasal 1 μg kg^−1^ dexmedetomidine induces appropriate sedation within 25–30 min and lasts for about 85 (55–100) min with only a modest reduction in heart rate (HR) and blood pressure (BP) (Yuen et al., 2010; Yuen et al., 2008). In our pilot study, 3 μg kg^−1^, but not 2 μg kg^−1^, of intranasal dexmedetomidine had significantly improved the behaviors in children during inhalation induction. However, a higher dose of dexmedetomidine would prolong the postoperative recovery time significantly, and it is less practical for shorter and minor same-day surgeries.

Numerous studies of sedation with intranasal dexmedetomidine only or oral midazolam only can be found in the literature. However, the investigational reports about the combination of these two drugs are very scanty in the pediatric population. Both medications suppress consciousness (Yoon et al., 2016) by acting on different receptors, and theoretically, their combination would enhance the sedative results, but not their adverse effects. Therefore, in our study, we compared the effects of both intranasal dexmedetomidine and oral midazolam, given either combined or separated, on the compliance of anesthesia inhalation induction and the process of preoperative sedation in children undergoing minor surgery.

## Methods

### Study Design

The prospective, randomized, double-blind study was performed after getting approved by the Ethics Committee of the Second Affiliated Hospital and Yuying Children’s Hospital of Wenzhou Medical University (Reference No. LCKY 2019-294, September 30, 2019) and registered at ClinicalTrials.gov (NCT 04135014, October 22, 2019). Written informed consent was obtained from the parents or legal guardians of pediatric patients.

### Study Population

#### Inclusion Criteria

In this study, 138 children, with American Society of Anesthesiologists (ASA) physical status I or II, aged 2–6 years, and within a normal range of weight, who were scheduled to undergo elective minor surgery (lower abdominal or perineal surgery with an expected operation time less than 30 min) were enrolled.

#### Exclusion Criteria

Children were excluded if they have gastrointestinal, cardiovascular, endocrine, or mental and developmental disorders, and have a known allergy to either dexmedetomidine or midazolam or have any other contraindications for preoperative sedation. Children would be disqualified for enrollment if having nasal pathology, recent upper respiratory infection (within 2 weeks), or any other reasons being considered as an inappropriate candidate at anesthesiologist’s discretion.

#### Data Collection

A case report form (CRF) was designed to record clinical data and was kept in a password-protected computer. Good clinical practice (GCP) was strictly followed during the study. Data were collected, filed, transferred by a specifically assigned researcher, and rechecked by another independent researcher to ensure its accuracy and safety.

#### Randomization and Blindness

In total, 138 children were randomly allocated (1:1:1 ratio) into three groups according to a computer-generated table: Group M to receive 0.5 mg kg^−1^ oral midazolam and intranasal saline, Group D to receive 2 μg kg^−1^ intranasal dexmedetomidine and oral saline, and Group M + D to receive 0.5 mg kg^−1^ oral midazolam plus 1 μg kg^−1^ intranasal dexmedetomidine. The group assignment was sealed in an envelope. Midazolam (5 mg ml^−1^, Jiangsu Enhua Pharma Corporation, China) was mixed with pear juice and sugar into a total volume of 5ml, and the undiluted and preservative-free dexmedetomidine (100 μg ml^−1^, Jiangsu Hengrui Pharma Corporation, China) was prepared in a 1 ml syringe prior to administration.

All study drugs were prepared by a designated researcher, and both the clinical data recorder and the attending anesthesiologists in the operating room were blinded to the study drug assignment.

#### Clinical Protocol

During the pre-anesthesia visit, the anesthetist conducted a routine medical evaluation on the child, instructed the parents or guardians with printed reading perioperative information, and encouraged them to visit the hospital website for more details. All children were directed to comply with the ASA fasting guideline on the day of surgery.

In the holding area and 30 min before the anesthesia induction, midazolam or equal volume of saline was given orally, and intranasal dexmedetomidine or an equal volume of saline was given through an atomizer (Teleflex MAD Nasal; Research Triangle Park, NC, United States) based on group assignment when parents were present. Standards for anesthesia monitoring included continuous electrocardiography, pulse oximetry, and every 5 min non-invasive BP recording. Anesthesia was induced with 8% sevoflurane in 100% oxygen at a flow rate of 6 L min^−1^. Peripheral venous access was established after exhalation sevoflurane had reached 2.0 MAC (minimal alveolar concentration) and adequate jaw relaxation was ensured, and then, a laryngeal mask airway (LMA) was inserted. An iliohypogastric/ilioinguinal nerve block with 6 ml of 0.15% ropivacaine + 0.8% lidocaine was implemented on the ipsilateral side for hernia or hydrocele repair, and a caudal block of 0.5 ml/kg of 0.15% ropivacaine +0.8% lidocaine was performed for circumcision or other urinary surgeries. Anesthesia depth was kept at 1.3–1.5 MAC of sevoflurane with 50% oxygen/air mixture, and spontaneous breath was maintained throughout the surgery. No other sedatives or opioids were administered. At the end of the surgery, the LMA was removed at 1.5 MAC of sevoflurane, and then, the sevoflurane was discontinued. The child was transferred to the post-anesthesia care unit (PACU).

The child was discharged from the PACU to the surgical ward after a modified Aldrete score reached 9.

### Assessment Parameters

#### Hemodynamics

Heart rate (HR), non-invasive BP, and pulse oximetry started recording just before the premedication (0 min as baseline) and then every 5 min till patients’ discharge from PACU. The episodes of hypotension (systolic BP < 70mmHg + (2 x age in years)) and bradycardia (HR < 70/min) were recorded.

#### UMSS

The sedation level was assessed using the University of Michigan Sedation Score (UMSS, Supplementary Table S1) every 10 min prior to surgery starting time. UMSS1 is defined as minimally sedated with an appropriate response to verbal and sound stimulations. UMSS2 is the moderate sedation with the patient lying somnolent, sleeping and easily aroused with a light touch. UMSS3 is defined as deep sedative status and arousable only with pain stimulation. UMSS4 is an unarousable status by any stimulation. Failed sedation was defined as UMSS＜2 within 30 min after the study drug was administered. The time required to achieve satisfactory sedation (UMSS ≥2) and the sedation failure rate were recorded.

#### *m*-YPAS, Behavior Score, and PSAS

The modified Yale Preoperative Anxiety Scale (*m*-YPAS) score was assessed after children were admitted to the holding area (Supplementary Table S2). The acceptance of premedication was evaluated with a 4-point behavior score when premeds were given (Supplementary Table S1). Parental separation was scored by a four-point parental separation anxiety scale (PSAS, S1).

#### Induction Compliance Checklist (ICC) and Mask Acceptance Scale (MAS)

ICC was accomplished by anesthesiologists in the operating room (Supplementary Table S3). This scoring system with a scale from 0 to 10 was designed by Kain et al. where the scores ≤3 signified satisfactory compliance, whereas scores ≥4 were classified as unsatisfactory compliance (Varughese et al., 2008; Lacquiere and Courtman, 2011). A four-point MAS (Supplementary Table S1) was performed at the beginning of anesthesia induction, and MAS one or two was considered as “satisfaction” to accept the face mask.

#### Pediatric Anesthesia Emergence Delirium Scale (PAED)

PAED is a reliable tool in the diagnosis of Emergence Delirium (ED) (Ringblom et al., 2018), and it consists of five items describing the behavior during the recovery periods (Supplementary Table S4). A higher score indicated more severe symptoms. Patients were considered having developed ED when PAED scores were ≥10, and a severe ED was defined when PAED scores were ≥15 (Hauber et al., 2015). For patients who developed delirium and could not be comforted, fentanyl 0.5 μg kg^−1^ or propofol 1 mg kg^−1^ was administered as a supplemental treatment.

#### Other Assessments

The total anesthesia time and the recovery time (from discontinuing anesthesia to discharge) were recorded. Postoperative nausea or vomiting, respiratory depression, and abnormal psychological/psychiatric behaviors were also collected.

### Statistical Analysis

#### Power of the Study

The primary outcome was the children’s compliance rate with anesthesia inhalation induction indicated by ICC and MAS scores. The secondary outcomes included the features of sedation assessed with UMSS score, preoperative anxiety by *m*-YPAS, behavior scores, and parental separation anxiety (PSAS) scores, perioperative HR and BP, and the incidence of ED. Our preliminary study showed that the satisfactory compliance with inhalation induction was approximately 45% in patients who were premedicated with oral midazolam alone. Hence, a sample size of 114 patients would provide 90% power at a 0.05 level of significance to detect a 10% difference of satisfactory compliance in each group. By estimating a potential 20% dropout, 138 children were needed in this study (G*Power).

#### Data Analysis

Patients who had a failed preoperative sedation were excluded from the study. The data were analyzed by SPSS version 24.0 for Windows (SPSS Inc., Chicago, IL, United States). The normality of the distribution of continuous variables was tested by a one-sample Kolmogorov–Smirnov test. Continuous variables with normal distribution were presented as mean ± standard deviation (SD), while non-normal variables were presented as median (interquartile range). Comparison of different groups was performed by one-way analysis of variance. Only if the ANOVA test was significant, the *p* value for pairwise comparisons was calculated using Student’s test with Bonferroni’s correction. Data are presented as the number for categorical variables. The intergroup difference was compared using the chi-square test or Fisher’s exact test for categorical variables. The baselines prior to premed administration were compared with the data after premedication by repeated-measures ANOVA. All statistical tests were two-sided, and a *p* value of <0.05 was considered significant.

## Results

**FIGURE 1:**
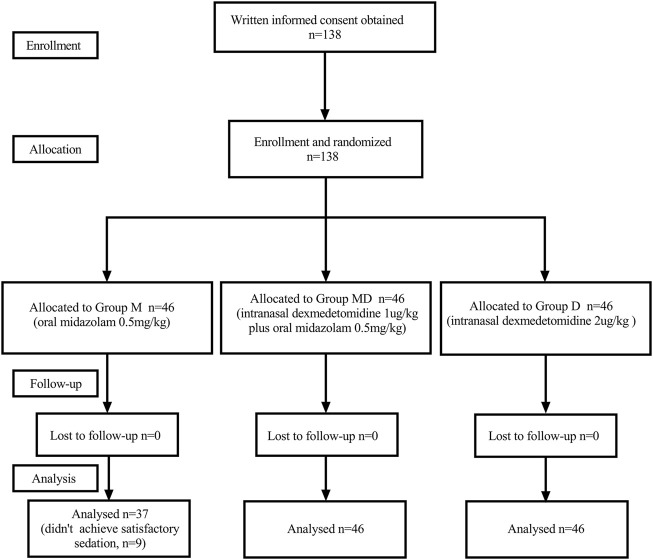
Consort flow diagram.

One hundred thirty-eight initially eligible children were randomly assigned to each group. Nine children in Group M were removed from the study later due to failed preoperative sedation (Figure 1). One hundred twenty-nine children had completely collected data for statistical analysis. There were no significant differences between groups in characteristics or baseline data. (Table 1).

**TABLE 1:** Subject characteristics and clinical data. Data are expressed as median and range, number, and frequency. PSAS, parental separation anxiety scale. *m*-YPAS, modified Yale Preoperative Anxiety Scale. Data are expressed as median (IQR [range]) or number (proportion).

	Group M oral midazolam (n = 37)	Group D intranasal dexmedetomidine (n = 46)	Group MD oral midazolam and intranasal dexmedetomidine (n = 46)	*P*
Age (yr)	5.1 (3.0,5.8)	4.3 (3.4,5.8)	5.0 (3.4,5.7)	0.9503
Median (interquartile range, IQR)				
Weight (kg)	19.0 (15.5,22.0)	18.0 (16.0,20.0)	18.0 (15.6,21.4)	0.9241
Median (interquartile range, IQR)				
Sex (male/female), n	34/3	41/5	41/5	0.9879
ASA physical status 1/2, n	37/0	45/1	46/0	0.9304
m-YPAS score	41.7 (31.7,50.0)	42.5 (36.7,51.7)	41.7 (36.7,56.7)	0.2448
Median (interquartile range, IQR)				
PSAS, n				
Excellent/good/fair/poor	27/7/2/1	38/3/3/2	39/4/2/1	0.8675
Acceptable separation	34	43	41	0.763
Type of surgery, n				0.3858
Hernia repair	13	17	18	
Peritomy	13	15	12	
Hydrocele	7	14	10	
Other	4	0	6	
Caudal anesthesia (yes/no), n	17	15	16	0.5649
Satisfactory sedation time (min)	24.0 (21.0, 26.0)	19.5 (18.0,23.0)*	15.0 (13.0,18.0)^#^	<0.0001
Median (interquartile range, IQR)				
Anesthesia time (min)	29.0 (23.0,41.0)	29.0 (22.5,38.0)	27.5 (21.5,40.8)	0.875
Median (interquartile range, IQR)				
Recovery time (min)	41.0 (28.0,61.0)	45.0 (31.0,61.0)	45.5 (35.0,58.0)	0.4065
Median (interquartile range, IQR)				
PAED, n: no/yes/severe	27/5/5	32/12/2	39/5/2	0.2391
Incidence of agitation	27.0%	30.4%	15.2%	0.7144

Compared with Group M, *p < 0.05, #*p* < 0.0001.

### Hemodynamics

Compared with Group M and after receiving premeds, children in Group D had significantly lower mean SBP and HR at 20–30 min (*p* < 0.01), significantly lower mean DBP at 15–30 min (*p* < 0.01), and lower MAP at 15–30 min (*p* < 0.01). Children in Group M + D had significantly lower MAP at 10–30 min after premeds (*p* < 0.01) than those in Group M. No other significant differences were found between groups at other time points (Supplementary Table S5). There was no incidence of defined bradycardia or hypotension recorded in all study groups.

### UMSS

**FIGURE 2:**
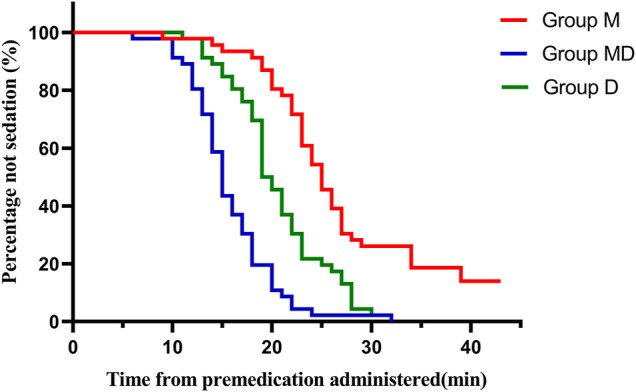
Onset time of sedation after drug administration. Patients in Group D and Group MD had significantly shorter satisfactory sedation time (*p* < 0.05) than those in Group M.

After receiving premeds, children in Group D and Group M + D had a significantly quicker onset to the satisfactory level of sedation (*p* < 0.05) (Table 1) and continued maintaining a higher sedation score (UMSS) at 20 and 30 min (*p* < 0.05) (Table 2 and Figure 2) than those in Group M.

**TABLE 2:** Sedation score (UMSS) after drug administration. Data are expressed as median and interquartile range (IQR).

	0 min	10 min	20 min	30 min
Group M	0 (0,0)	0 (0,1)	1 (1,1)	2 (2,2)
Group D	0 (0,0)	0 (0,1)	2 (1,3)*	3 (2,3)^#^
Group MD	0 (0,0)	0 (0,1)	2 (2,3)^#^	3 (2,3)^#^

Compared with Group M, *p < 0.05, #*p* < 0.0001).

### *m*-YPAS, Behavior Score, and PSAS

There were no significant differences among the groups in terms of *m*-YPAS (Table 1), behavior scores, and PSAS (Table 3).

**TABLE 3:** Preoperative assessments (PSAS, parental separation anxiety scale; Behavior Score); postoperative PAED assessment (PAED, Pediatric Anesthesia Emergence Delirium). Behavior scores with drug administration, compliance during induction of anesthesia, and Mask acceptance scale (MAS). ICC, induction compliance checklist.

	Group M oral midazolam (n = 37)	Group D intranasal dexmedetomidine (n = 46)	Group MD oral midazolam and intranasal dexmedetomidine (n = 46)	*P*	Compared group	*P*
Behavior score, n				0.332	-	-
1	10	9	7		-	-
2	10	15	18		-	-
3	13	9	12		-	-
4	4	13	9		-	-
ICC score, n				0.003	-	-
0 (perfect)	6	6	20	-	Group M vs. D	1.000
1–3 (moderate)	14	15	18	-	Group MD vs. D	<0.001
≥4 (poor)	17	25	8	-	Group MD vs. M	0.004
Satisfactory compliance, n<	20^+^	21^+^	38	0.0049	-	-
MAS				0.016	-	-
Excellent	9	11	26	-	Group M vs. D	1.000
Good	11	10	12	-	Group MD vs. D	0.001
Fair	11	14	3	-	Group MD vs. M	0.012
Poor	6	11	5	-	-	-
Satisfactory MAS, n	20^+^	21^+^	38	0.0049	-	-

Compared with Group MD, *P<0.05

### ICC and MAS Assessments

ICC scores in Groups M and D were significantly lower than those in the M + D Group (54.1% and 45.7 vs. 82.6%; *p* < 0.05, Table 4), and no significant differences was found between Groups M and D. When ICC scores were graded into perfect, moderate, and poor compliance levels, the numbers of patients in each level were 20, 18, and 8 in the M + D group, 6, 15, and 25 in the D group ((*p* < 0.001, compared with Group M + D), and 6, 14, and 17 in the M group (*p* = 0.004, compared with Group M + D). Children in the M and D Group had lower MAS scores (*p* = 0.012, *p* = 0.001) and lower satisfactory MAS (*p* < 0.05) (Table 4) than patients in Group M + D. There were no significant differences between the groups in terms of anesthesia time and recovery time (Table 1).

**TABLE 4:** Vomiting, Excessive salivation and Psychological/psychiatric events.

	Group M (n = 37)	Group D (n = 46)	Group M+D (n = 46)	P
Vomiting	1	0	0	0.773
Excessive salivation	2	0	1	1.000
Psychological/psychiatric events	0	0	1	1.000

### PAED and Other Assessments

There were no significant differences in the incidence of PAED among groups (Table 3). The numbers of patients who developed postoperative vomiting were 1, 0, and 0 in Groups M, D, and M + D, respectively. No airway events and no episodes of hypoxia were observed (Table 5).

**TABLE 5:** Vomiting, excessive salivation, and psychological/psychiatric events.

	Group M (n = 37)	Group D (n = 46)	Group M + D (n = 46)	P
Vomiting	1	0	0	0.773
Excessive salivation	2	0	1	1.000
Psychological/psychiatric events	0	0	1	1.000

## Discussion

In this study, we found that children who were premedicated with intranasal dexmedetomidine combined with oral midazolam had a significantly improved compliance rate during anesthesia inhalation induction and had faster onset to achieve the desired sedation than those premedicated only with intranasal dexmedetomidine or oral midazolam group.

It has been well known that preoperative sedation could reduce anxiety in pediatric patients and facilitate a smooth anesthesia induction (Yuen et al., 2012; Kumari et al., 2017). Midazolam is one of the most popular premeds, and its recommended oral dose is 0.5 mg kg^−1^ (Kumari et al., 2017). Intranasal dexmedetomidine was another widely used premed (Talon et al., 2009; Yuen et al., 2012), and it showed reasonable sedative effect without increasing untoward reactions when the dosage was set at 2 μg kg^−1^ (Yuen et al., 2012). However, none of those individual pharmacological agents could have completely fulfilled the clinical expectations in children undergoing surgeries (Kain et al., 2004a; Kain et al., 2004b; Davidson and McKenzie, 2011). There have been reports of using combined intravenous dexmedetomidine and midazolam in adult patients, but few were found in pediatric studies. Therefore, we combined these two premeds together and applied them to the same surgical pediatric patient, while oral midazolam only or intranasal dexmedetomidine only was used as a separate control group. Results showed that the combined premeds of oral midazolam and intranasal dexmedetomidine facilitated the onset of sedation and improved the smoothness of anesthesia inhalation induction compared to the non-combined premed technique. There have been some concerns about the side effects caused by either midazolam or dexmedetomidine, such as paradoxical reaction, prolonged recovery, hypotension, and bradycardia, and let alone when both premeds are given together. For that reason, we decided to use 1 μg/kg intranasal dexmedetomidine in the study group instead of 2 μg/kg as in the control group. Our study results did not show any significant increase of the undesired effects and there were no differences in adverse effects among the three groups.

ICC, which has high accuracy and objectivity to evaluate the degree of cooperativeness, was used to assess the smoothness of anesthesia inhalation induction, while MAS was used to assess the reaction to accept a face mask during inhalation induction. A study showed that 37.5% of pediatric patients out of those premedicated with 0.5 mg kg^−1^ oral midazolam had satisfactory ICC scores (Sadeghi et al., 2017) when parents were not present during anesthesia induction. Sathyamoorthy et al. used MAS to evaluate children who received 0.5 mg kg^−1^ oral midazolam, and they found that the rate of willingness to accept face mask inhalation induction was 78%, which was similar to the results prompted by giving intranasal 2 μg kg^−1^ dexmedetomidine (Sathyamoorthy et al., 2019). In our study, when the two premeds were applied together to the same patient, more satisfactory anesthesia induction compliance checklist (ICC) and face mask acceptance (MAS) were observed than that from a single premed. There were no significant differences in ICC and MAS between Group M and Group D in our study, which was in agreement with Sathyamoorthy’s research.

The optimal regimen of premedication should have a quicker onset, which could cut the waiting time, help alleviate parents’ anxiety, and reduce negative pain memories of children (Fischer et al., 2019). It should also have a relatively short sedation duration, which facilitates patient’s recovery. A previous study found that the time to achieve satisfactory sedation is approximately 20–30 min (Lammers et al., 2002; Somri et al., 2012) for oral midazolam and about 25 min for intranasal dexmedetomidine (Yuen et al., 2010). In a study by Lin et al., the recovery time of patients receiving intranasal 2 μg kg^−1^ dexmedetomidine was 20 min (Lin et al., 2016). Therefore, we administered premeds 30 min before the surgery starting time, and the results showed that the average onset time to the desired sedation in the combined premeds group (M + D) was only 15 min, which was significantly shorter than that in Group M. Our study did not show significant differences in postoperative recovery times among three groups, and the discharge time was not delayed in the M + D group. It could be hypothesized that the sedative effect of premeds had largely faded off through the surgeries at the time patients had arrived at PACU.

ED is a common complication in children after sevoflurane anesthesia. The incidence of ED in preschool children varies greatly from 10 to 80% (Lin et al., 2016), subject to assessments of rating scales or criteria. Pediatric Anesthesia Emergence Delirium Scale has been widely used in many studies to evaluate the ED occurrence, and the diagnosis of ED can be made when PAED scores ≥10 or 12 (Hauber et al., 2015). Guler et al. found that intravenous administration of 0.5 μg kg^−1^ dexmedetomidine for 5 min had decreased the incidence of ED from 57 to 17% (Guler et al., 2005). Shukry et al. demonstrated that a continuous infusion of 0.2 μg kg^−1^ h^−1^ dexmedetomidine could reduce the incidence of ED from 61 to 26% (Shukry et al., 2005). However, the effect of midazolam on the occurrence of ED remains controversial. A cohort study by Maeda and others showed that oral midazolam was even an independent risk factor for pediatric postoperative agitation. In our study, the incidence of ED of Group M + D is 15.2% (Maeda et al., 2012), and there were no differences when compared with Group M or Group D. As we have mentioned previously, the effects of either midazolam or dexmedetomidine on ED could have been nearly worn off after 30 min premed plus 30 min surgical time. It is noticeable that premeds should be given close to the end of anesthesia and surgeries to have an impact on the occurrence of ED as reported by many studies.

Midazolam usually does not cause negative hemodynamic changes, especially in an oral form at a dosage of 0.5 mg kg^−1^, and by contrast, dexmedetomidine might induce undesired hypotension and bradycardia without significant respiratory depression (Plambech and Afshari, 2015). Yuen et al. reported that HR and systolic BP will decrease by 16.4 and 14.1%, respectively, after patients received 1 μg kg^−1^ intranasal dexmedetomidine (Yuen et al., 2007). In our study, the combined regimens (oral midazolam 0.5 mg kg^−1^ plus intranasal dexmedetomidine 1 μg kg^−1^) did not show significant hemodynamic changes but did have fewer episodes of decreased HR and BP than the 2 μg kg^−1^ dexmedetomidine group in the holding area. Treatment was not rendered since the hemodynamic changes were insignificant.

Other combined premed regimens have been tried in children. Oral low-dose ketamine plus intranasal dexmedetomidine can produce satisfactory sedation and facilitate smooth venous cannulation without seeing excessive side effects or postoperative complications (Jia et al., 2013; Qiao et al., 2017). However, the high incidence of hallucinations, delirium, copious secretion, and postoperative agitation, nausea, and vomiting has limited ketamine’s clinical applications (Gingrich, 1994). By contrast, oral midazolam was considered as a safer premed, and Li et al. (Li et al., 2018) advocated that a combination of intranasal dexmedetomidine and buccal midazolam resulted in deeper sedation with a higher rate of sedation success than chloral hydrate or dexmedetomidine alone. The study also suggested that intranasal dexmedetomidine-midazolam is a safe and effective method to achieve moderate sedation in children (Fett et al., 2017). Although the intranasal route is a convenient way with higher bioavailability, it may not always be easily accepted by some kids because of its burning sensation to the nasal mucosa.

### Limitation

There are a few limitations. First, due to the anatomic and surgical nature, the proportion of male patients was larger than that of female patients in this study, which might potentially have skewed the final results due to the gender distribution imbalance. Secondly, no further follow-up was performed after patients’ discharge to home; we could miss the information of long-term effect on the children’s behavior and cognition.

## Conclusion

Children premedicated with combined intranasal dexmedetomidine 1 μg kg^−1^ and oral midazolam 0.5 mg kg^−1^ had shorter onset to the desired satisfactory sedation and had a higher compliance rate during anesthesia inhalation induction than those premedicated with either intranasal dexmedetomidine or oral midazolam alone.

## Data Availability

The raw data supporting the conclusion of this article will be made available by the authors, without undue reservation.

## Abbreviation

ASA, American Society of Anesthesiologists; BP, blood pressure; CRF, case report form; DBP, non-invasive diastolic blood pressure; ED, emergence delirium; GCP, Good Clinical Practice; HR, heart rate; ICC, induction compliance checklist; LMA, laryngeal mask airway; MAC, minimal alveolar concentration; MAS, mask acceptance scale; MAP, mean arterial pressure; *m*-YPAS scores, the modified Yale Preoperative Anxiety Scale; OR, operating room; PACU, postanesthesia care unit; PAED, Pediatric Anesthesia Emergence Delirium Scale; PSAS, Parental Separation Anxiety Scale; SBP, non-invasive systolic blood pressure; UMSS, University of Michigan Sedation Score
