# Supplementary material for: Comparison of the Effects of Oral Midazolam and Intranasal Dexmedetomidine on Preoperative Sedation and Anesthesia Induction in Children Undergoing Surgeries
Source: Front Pharmacol. 2021 Dec 15;12:648699. doi: 10.3389/fphar.2021.648699 (PMC8714926; doi:10.3389/fphar.2021.648699)
Supplement: Supplementary file 1 [file DataSheet1.doc]

**Table S1** Evaluation Scale

UMSS: the sedation scale of the University of Michigan; PSAS: Parental separation anxiety scale

| Behavior scores |  |
| --- | --- |
| 1 | Clam and cooperative |
| 2 | Anxious but reassurable |
| 3 | Anxious but not reassurable |
| 4 | Crying, or resisting |
| UMSS |  |
| 0 | Awake and alert |
| 1 | Minimally sedated: tired/sleepy, appropriate response to verbal conversation and/or sound |
| 2 | Moderately sedated: somnolent/sleeping, easily aroused with light tactile stimulation or a simple verbal command |
| 3 | Deeply sedated: deep sleep, arousable only withsignificantphysical stimulation |
| 4 | Unarousable |
| PSAS |  |
| 1 | Crying, very anxious |
| 2 | Anxious, not crying |
| 3 | Calm, but not cooperative |
| 4 | Calm, cooperative or asleep |
| MAS |  |
| poor | terrified, crying, agitated |
| fair | moderate fear, not calmed with reassurance |
| good | slight fear of mask, easily calmed |
| excellent | unafraid, cooperative, accepts mask readily |

MAS: Mask acceptance scale

**Table S2** mYPAS score

| categories | items | scores |
| --- | --- | --- |
| Activity | Looking around, curious, playing with toys, reading (or other age-appropriate behavior); moves around holding area/treatment room to get toys or to go to parent; may move toward operating room equipment | 1 |
|  | Not exploring or playing, may look down, fidget with hands, or suck thumb (blanket); may sit close to parent while waiting, or play has a definite manic quality | 2 |
|  | Moving from toy to parent in unfocused manner, non-activity-derived movements; frenetic/frenzied movement or play; squirming, moving on table; may push mask away or cling to parent | 3 |
|  | Actively trying to get away, pushes with feet and arms, may move whole body; in waiting room, running around unfocused, not looking at toys, will not separate from parent, desperate clinging | 4 |
| Vocalizations | Reading (nonvocalizing appropriate to activity), asking questions, making comments, babbling, laughing, readily answers questions but may be generally quiet; child too young to talk in social situations or too engrossed in play to respond | 1 |
|  | Responding to adults but whispers, “baby talk,” only head nodding | 2 |
|  | Quiet, no sounds or responses to adults | 3 |
|  | Whimpering, moaning, groaning, silently crying | 4 |
|  | Crying or may be screaming “no” | 5 |
|  | Crying, screaming loudly, sustained (audible through mask) | 6 |
| Emotional expressivity | Manifestly happy, smiling, or concentrating on play | 1 |
|  | Neutral, no visible expression on face | 2 |
|  | Worried (sad) to frightened, sad, worried, or tearful eyes | 3 |
|  | Distressed, crying, extreme upset, may have wide eyes | 4 |
| State of apparent arousal | Alert, looks around occasionally, notices or watches what anesthesiologist does (could be relaxed) | 1 |
|  | Withdrawn, sitting still and quiet, may be sucking on thumb or have face turned into adult | 2 |
|  | Vigilant, looking quickly all around, may startle to sounds, eyes wide, body tense | 3 |
|  | Panicked whimpering, may be crying or pushing others away, turns away | 4 |
| Use of parents | Busy playing, sitting idle, or engaged in age-appropriate behavior and doesn’t need parent; may interact with parent if parent initiates the interaction | 1 |
|  | Reaches out to parent (approaches parent and speaks to otherwise silent parent), seeks and accepts comfort, may lean against parent | 2 |
|  | Looks to parent quietly, apparently watches actions, doesn’t seek contact or comfort, accepts it if offered or clings to parent | 3 |
|  | Keeps parent at distance or may actively with-draw from parent, may push parent away or desperately clinging to parent and not let parent go | 4 |

**Table S3** Induction compliance checklist ( ICC)

| item | score |
| --- | --- |
| Crying, tears in eyes | 1 |
| Turns head away from mask | 1 |
| Verbal refusal, says “no,” | 1 |
| Verbalization indicating fear or worry, “where’s mommy?” or “will it hurt?” | 1 |
| Pushes mask away with hands, pushes nurse or anesthesiologist with hands/feet | 1 |
| Covers mouth/nose with hands/arms or buries face | 1 |
| Hysterical crying, may scream | 1 |
| Kicks/flails legs/arms, arches back, and/or general struggling | 1 |
| Requires physical restraint | 1 |
| Complete passivity, either rigid or limp | 1 |
| perfect induction (does not exhibit negative behaviors, fear or anxiety | 0 |

**Table S4** Pediatric anesthesia emergence delirium scale (PAED)

| Behavior | | Not at all | | Just a little | | Quite a bit | | Very much | | Extremely |
| --- | --- | --- | --- | --- | --- | --- | --- | --- | --- | --- |
| Makes eye contact | | 4 | | 3 | | 2 | | 1 | | 0 |
| Actions are purposeful | 4 | | 3 | | 2 | | 1 | | 0 | |
| Aware of surroundings | 4 | | 3 | | 2 | | 1 | | 0 | |
| Restless | 0 | | 1 | | 2 | | 3 | | 4 | |
| Inconsolable | 0 | | 1 | | 2 | | 3 | | 4 | |

1—Calm; 2—not calm but could be easily consoled; 3—moderately agitated or restless and not easily calmed; 4—combative, excited, thrashing around.

**Table S5** Heart rate (HR), systolic (SBP) and diastolic (DBP) after drug administration. P<0.05: significance vs Group M. Data are expressed as mean ± standard deviation (SD).

|  | 0min | | 5min | 10min | 15min | 20min | 25min | 30min |
| --- | --- | --- | --- | --- | --- | --- | --- | --- |
| SBP: | |  |  |  |  |  |  |  |
| Group M | | 107.4±11.42 | 104.1±8.77 | 102.1±10.29 | 98.9±9.53 | 99.1±8.99 | 99.4±11.60 | 97.5±7.93 |
| Group D | | 106.2±11.64 | 103.2±12.15 | 99.3±9.73 | 95.2±9.49 | 91.2±9.65^***^ | 90.1±9.87^**^ | 88.7±10.26^#^ |
| Group MD | | 104.1±11.42 | 101.4±12.19 | 97.4±10.47 | 96.4±10.60 | 95.3±10.62 | 94.2±8.30 | 92.7±7.31^*^ |
| DBP: | |  |  |  |  |  |  |  |
| Group M | | 60.0±9.75 | 59.2±10.35 | 68.1±11.24 | 57.5±9.73 | 54.8±9.51 | 54.8±9.79 | 54.7±9.19 |
| Group D | | 58.9±11.99 | 58.2±10.08 | 53.3±9.25 | 49.8±9.98^**^ | 48.0±10.57^**^ | 45.8±8.98^***^ | 45.1±7.69^#^ |
| Group MD | | 58.4±7.81 | 57.8±9.18 | 52.7±9.73 | 52.9±10.6 | 52.9±8.99 | 50.8±8.43 | 50.5±8.15 |
| MAP: | |  |  |  |  |  |  |  |
| Group M | | 75.8±8.95 | 74.1±8.88 | 72.8±9.53 | 71.2±8.35 | 69.5±7.89 | 69.6±9.41 | 69.0±7.78 |
| Group D | | 74.6±10.66 | 73.5±9.5 | 68.7±8.07^*^ | 65.0±8.71^***^ | 62.4±9.46^#^ | 60.6±8.83^#^ | 59.6±7.84^#^ |
| Group MD | | 73.7±7.62 | 72.4±8.63 | 67.7±8.10^**^ | 67.5±9.56^*^ | 67±8.27 | 65.3±7.47^*^ | 64.6±6.93^*^ |
| HR: | |  |  |  |  |  |  |  |
| Group M | | 98.4±14.21 | 97.1±13.2 | 95.3±13.78 | 95.5±14.28 | 95.5±13.36 | 96.6±14.38 | 95.5±15.16 |
| Group D | | 100.7±11.50 | 97.9±10.85 | 95.1±9.35 | 89.6±12.05 | 86.3±11.5^**^ | 83.4±11.14^#^ | 82.2±10.22^#^ |
| Group MD | | 98.2±12.12 | 95.0±12.73 | 94.0±12.45 | 91.6±13.35 | 90.3±12.94 | 90.2±12.26 | 89.4±11.65 |

compared with Group M, **P*<0.05, ***P*<0.01, ****P*<0.001, #*P*<0.0001
